# Determinants of measles-rubella second dose vaccine uptake among children in Mathare informal settlement, Nairobi, Kenya: a mixed-methods study

**DOI:** 10.11604/pamj.2026.53.43.49226

**Published:** 2026-01-29

**Authors:** Kenneth Kamande, Micah Matiang'i, Jeanette Dawa

**Affiliations:** 1 Department of Community Health, Amref International University, Nairobi, Kenya,; 2 Center for Epidemiological Modelling and Analysis, University of Nairobi, Nairobi, Kenya

**Keywords:** Immunization, vaccination, caregiver, measles, rubella

## Abstract

**Introduction:**

measles-rubella remains a major public health concern, with vaccination as the most effective preventive strategy. Despite the introduction of the measles-rubella second dose (MR-2) vaccine to improve immunity, uptake in informal settlements like Mathare, Nairobi, remains suboptimal. This study assessed determinants of MR-2 uptake among children aged 18-59 months in Mathare.

**Methods:**

this cross-sectional analytical study used a convergent mixed- methods design. Quantitative data were collected through structured household surveys, while qualitative insights were obtained from Key Informant Interviews and Focus Group Discussions.

**Results:**

a total of 370 caregivers responded (103% of the targeted 359). MR-2 coverage was 84%, 14% lower than the measles-rubella first dose (MR-1). Uptake was significantly associated with religion, education level, ward of residence, place of delivery, immunization site, and previous unsuccessful vaccination attempts (p < 0.05). Caregivers unaware of the MR-2 schedule were over four times more likely to miss the dose (OR = 4.146, p < 0.001), as well as those who had previously failed to access services (OR = 4.215, p = 0.007). Lack of a vaccination card (OR = 0.314, p = 0.015) and poor schedule knowledge (OR = 0.322, p = 0.014) were also key predictors. Children in Kiamaiko ward (OR = 5.421) and Ngei ward (OR = 4.281) had higher odds of MR-2 uptake compared to Mlango Kubwa ward. Qualitative findings highlighted barriers such as low awareness, misinformation, economic hardship, and health system gaps.

**Conclusion:**

improving MR-2 uptake requires enhanced health education, mobile reminders, consistent vaccine supply, extended clinic hours, and targeted outreach.

## Introduction

Measles and rubella are highly contagious viral diseases with serious public health consequences, particularly for children. Measles can lead to life-threatening complications, including pneumonia, encephalitis, and death, with a case fatality rate ranging from 0.1% in developed countries to 15% in Kenya [1]. Rubella, while typically less severe in children, poses significant risks to pregnant women, often leading to Congenital Rubella Syndrome (CRS), which causes severe birth defects [2].

Vaccination is the most effective strategy for preventing both diseases. The World Health Organization (WHO) estimates that immunization prevents 2.5 million deaths annually among children under five [3]. In Kenya, the combined measles-rubella (MR) vaccine was introduced into the routine immunization schedule in 2016, building on earlier measles vaccination efforts, with the second dose (MR-2) added in 2013 to ensure sustained immunity and protection among children who may not have developed adequate immunity after the first dose (MR-1) [4]. The second dose is critical for increasing population-level immunity by closing immunity gaps caused by primary vaccine failure or missed and delayed opportunities for vaccination. Despite these efforts, the uptake of MR-2, administered at 18 months of age, remains suboptimal, particularly in informal settlements like Mathare [5]. Mathare is one of the largest informal settlements in Nairobi City County, Kenya, characterized by high population density, poverty, inadequate housing, limited access to basic services, and overstretched public health facilities. These structural and socio-economic challenges contribute to poor health outcomes, including low immunization coverage and recurrent outbreaks of vaccine-preventable diseases.

In Mathare, MR-2 vaccination coverage among children aged 18-59 months is estimated at 37.2%, significantly lower than the national average of 50% [6]. This low MR-2 coverage indicates that fewer than four in ten eligible children in Mathare receive the recommended second dose of the measles-rubella vaccine. This gap in vaccine coverage exposes the population to preventable outbreaks, especially among children under five, exacerbated by factors such as socio-economic status, limited access to health services, and gaps in knowledge of immunization schedules [7]. This study was guided by the Andersen Behavioral Model of Health Service Utilization, which suggests that utilization of health service is determined by predisposing factors (such as caregiver's age, education, and knowledge and perceptions), enabling factors (including household's socio-economic status, access to health facilities, and availability of services), and need-related factors (perceived importance of vaccination and prior child illness). This framework is appropriate for understanding MR-2 uptake in informal urban settings, where individual behaviors interact with structural and health system barriers to influence immunization outcomes. The key variables explored in this study include caregiver socio-demographic characteristics, caregiver awareness and knowledge of MR-2, household socio-economic factors, and health facility-related factors such as accessibility, availability of services, and provider interactions.

Despite the availability of the measles-rubella vaccine in Kenya's routine immunization program, MR-2 coverage remains unacceptably low in urban informal settlements such as Mathare. This persistent gap threatens measles and rubella elimination efforts and disproportionately exposes vulnerable children to preventable disease and death. However, evidence on the combined influence of caregiver, household, and health system factors on MR-2 uptake in these settings remains limited. This study, therefore, aimed to identify the factors influencing MR-2 uptake among children aged 18-59 months in Mathare. By exploring demographic, socio-economic, and health facility-related determinants, the study seeks to provide evidence-based recommendations to improve vaccine coverage and reduce health inequities in Nairobi's informal settlements. Addressing these challenges will be critical in advancing the goal of measles and rubella elimination in Kenya.

## Methods

**Study design and location:** this study cross-sectional analytical study using a convergent mixed method design, integrating both quantitative and qualitative approaches, and was conducted in Mathare Sub-County, Nairobi County. In the convergent design, quantitative and qualitative data were collected simultaneously, analyzed separately, and then integrated during interpretation to provide a comprehensive understanding of MR-2 vaccine uptake determinants.

The quantitative component involved a household-based survey of caregivers of children aged 18-59 months to assess MR-2 vaccine uptake and associated demographic, socio-economic, and health service-related factors. Potential confounding variables, including caregiver age, education level, household socio-economic status, and child age, were included in multivariate logistic regression models to estimate adjusted odds ratios for determinants of MR-2 uptake.

The qualitative component included in-depth interviews and key informant interviews with caregivers, health workers, and community health volunteers to explore contextual, behavioral, and health system factors influencing MR-2 uptake. Qualitative rigor was ensured through multiple strategies: interviews were conducted by a trained team, data were triangulated across different participant groups, an audit trail of coding and analytic decisions was maintained, and emerging findings were validated with select participants to enhance trustworthiness and credibility. Mathare Sub-County consists of five wards: Huruma, Kiamaiko, Mabatini, Mathare, and Mlango Kubwa.

**Inclusion and exclusion criteria:** the study included caregivers of children aged 18-59 months who had lived in Mathare Sub-County for at least three months and provided informed consent. Caregivers of children with mental disabilities or those residing in Mathare for less than three months were excluded from the study, as were those who did not consent to participate.

**Sample size and power calculation:** using the Cochrane formula, the sample size for the household survey was calculated based on an MR-2 vaccination coverage rate of 37.2% (p = 0.372) from 2020 DHIS2 data, which is the proportion of interest (p), while q was 0.628.


$$n_{0}=\frac{1.96^{2}*0.372*0.628}{0.05^{2}}$$

$$n_{0}=\frac{3.8416*0.233616}{0.0025}$$

The quantitative sample size was 359 based on the expected MR-2 coverage, confidence level, and margin of error. A total of 370 caregivers were ultimately interviewed, slightly exceeding the calculated sample to account for potential non-response and ensure adequate representation.

**Sampling procedure:** the study employed a multi-stage sampling procedure. Mathare Sub-County comprises five wards, Huruma, Kiamaiko, Mabatini, Mathare, and Mlango Kubwa, each made up of several villages. A list of villages and their population sizes was obtained from Nairobi City County administrative records and Community Health Unit (CHU) registers, which served as the sampling frame. In Stage 1, villages (primary sampling units) were selected using Population Proportional to Size (PPS) to ensure a representative distribution of wards. In Stage 2, household sampling frames were developed using CHU household registers, supplemented by input from Community Health Volunteers and local leaders. The required number of households per village was allocated proportionally to village size, and systematic sampling was used to select households based on a calculated sampling interval and a random starting point. Where more than one eligible child was present, one child was selected at random.

**Data collection:** a mixed-methods approach was used for data collection.

***Quantitative data:*** structured questionnaires were administered to caregivers through trained enumerators to gather demographic, socio-economic, and vaccination history data. The questionnaire was adapted from the UNICEF/WHO/American Red Cross Expanded Programme on Immunization (EPI) survey tools and tailored to the local context. To ensure reliability and validity, a pilot test was conducted in a similar community within Mathare Sub-County. Adjustments were made to improve clarity and standardize administration. Real-time data entry was done using KOBO Collect.

***Qualitative data:*** key Informant Interviews (KIIs) with health facility representatives and county health officials, as well as Focus Group Discussions (FGDs) with caregivers, were conducted to explore perceptions and experiences of MR-2 vaccination. Qualitative facilitators and interviewers received structured training on conducting interviews and focus groups, probing techniques, and ethical considerations. Reflexivity was addressed by having researchers indicate their assumptions and positionality, ensuring awareness of potential biases during data collection and analysis.

**Data analysis:** before the main study, the data collection tools (questionnaires and interview guides) were pretested in a similar community in Mathare Sub-County to ensure clarity, accuracy, and appropriateness. Adjustments were made based on feedback from the pretest to improve reliability.

***Quantitative data:*** descriptive statistics (percentages and frequencies) were used to summarize demographic information and MR-2 vaccination coverage. Univariate and multivariate analyses in R programming software were employed to identify determinants of vaccine uptake. Univariate logistic regression was first conducted to examine the association between each independent variable and MR-2 uptake. Variables with p < 0.2 in the univariate analysis were included in the multivariable logistic regression model to control for potential confounders. Adjusted Odds Ratios (aORs) with 95% Confidence Intervals (CIs) were reported, and p-values < 0.05 were considered statistically significant. All analyses were performed in R programming software.

***Qualitative data:*** transcripts from KIIs and FGDs were coded and analyzed thematically. A coding framework was developed based on the study objectives and initial reading of transcripts. Two researchers independently coded a subset of transcripts to ensure consistency, and discrepancies were resolved through discussion. NVivo software was used for organizing and managing qualitative data, allowing for an in-depth exploration of the factors influencing vaccination behavior. The qualitative findings were triangulated with the quantitative results for a more comprehensive understanding.

**Ethical approval:** it was granted by the Amref Ethics and Scientific Review Committee (ESRC) Ref. AMREF-ESRC P1361/2022 and a research permit was obtained from the National Commission for Science, Technology, and Innovation (NACOSTI) License no. NACOSTI/P/23/24265. Informed consent was obtained from all participants, and confidentiality was maintained throughout the study.

## Results

**Demographic characteristics:** a total of 370 caregivers of children aged 18-59 months participated in the study. The majority were female (91%), aged 18-35 years (75%), and married (68%). Most had attained secondary (60%) or primary education (24%), and 85% had one eligible child. Awareness level was high, with (93%) being aware of the MR vaccine, and 91% possessing a child health card. Overall, 98% of children had received the first MR dose, MR-2 uptake dropped to 84%, indicating that 16% of children missed MR-2. MR-2 uptake varied significantly by ward of residence (p < 0.001). Mlango Kubwa Ward had the highest MR-2 uptake at 93%, followed by Huruma at 91%. Mabatini (83%) and Kiamaiko (82%). The lowest uptake was recorded in Ngei Ward at 39%. Caregiver religion was significantly associated with MR-2 uptake (p = 0.045), with higher uptake observed among Muslim and other religious groups (100%) compared to Christians (83%). Caregiver age, education level, gender, and marital status were not significantly associated with MR-2 uptake (p > 0.05).

**Social and economic factors:** approximately 43% of caregivers were unemployed, 46% self-employed, and only 11% formally employed. Among the employed, 40% held semi-skilled jobs. Household incomes were low, with 41% earning between Ksh. 1,000-5,000 per month. Although financial barriers were reported in focus group discussions (FGDs), caregiver occupation (p = 0.498) and household income (p = 0.489) were not statistically associated with MR-2 uptake. Economic constraints, especially the risk of lost income from informal work, were commonly cited as reasons for missed appointments (Table 1, Table 2).

**Table 1:** measles-rubella second dose (MR-2) uptake association

Demographic factors					
		**Received MR2 vaccine**	**Did Not Receive MR2 vaccine**	**Total**	**Chi square p value**
Age of the caregiver	18-35 Years	83%	17%	**100%**	.798a,b
	36-49 Years	86%	14%	**100%**	
	50-64 Years	100%	0%	**100%**	
Caregivers’ education	No formal education	100%	0%	**100%**	**.057a,b**
	Primary	87%	13%	**100%**	
	Secondary	86%	14%	**100%**	
	Tertiary and above	70%	30%	**100%**	
Caregivers’ religion	Christian	83%	17%	**100%**	.045*,b,c
	Muslim	100%	0%	**100%**	
	Other	100%	0%	**100%**	
Marital status of the caregiver	Married	84%	16%	**100%**	.992a,b
	Single	88%	12%	**100%**	
	Separated/ Divorced	85%	15%	**100%**	
	Widowed	83%	17%	**100%**	
Ward	Huruma	91%	9%	**100%**	.000*
	Kiamaiko	82%	18%	**100%**	
	Mabatini	83%	17%	**100%**	
	Mlango Kubwa	93%	7%	**100%**	
	Ngei	39%	61%	**100%**	
				**100%**	
**Socio-economic factors**					
Household Income	Less than Ksh. 1000	76%	24%	**100%**	0.489
	Ksh. 1000-5000	88%	12%	**100%**	
	Ksh. 5001-10,000	83%	17%	**100%**	
	More than Ksh. 10,000	81%	19%	**100%**	
				**100%**	
**Health facility-related factors**					
Sought the MR vaccine services but not attended to	Yes	95%	5%	**100%**	.016*
	No	81%	19%	**100%**	
Attended ANC during pregnancy	Yes	84%	16&	**100%**	.351a,b
	No	50%	50%	**100%**	
Place of Delivery	Home	71%	29%	**100%**	**.050a,b**
	Health facility	84%	16%	**100%**	
Knows about the MR Vaccines	Yes	84%	16%	**100%**	.152a
	No	85%	15%	**100%**	
Knows MR vaccine administration in two doses at 9 and 18 months	Yes	86%	14%	**100%**	.000*
	No	64%	36%	**100%**	
Point of immunization	Public/government facility	83%	17%	**100%**	.000*,b,c
	Private facility	86%	14%	**100%**	
	Faith Based Organization	100%	0%	**100%**	
	Other	50%	50%	**100%**	
	Don’t Know	100%	0%	**100%**	

* : statistically significant association (p < 0.05); a: one or more cells have expected counts < 5; b: more than 20% of cells have expected counts <5; c: minimum expected cell count <1

**Table 2:** summary of the reasons for not taking measles-rubella (MR) vaccine

Reasons for not taking MR Vaccine	Freq. (n)	Perc. (%)
Mother too busy	121	33%
Fear of side effects of the vaccine	94	25%
Long waiting time	80	22%
Place and/or time of immunization unknown	53	14%
Time of immunization not convenient	51	14%
Religion	49	13%
Traditions and cultural values	48	13%
Family problem, illness of mother	44	12%
Place of immunization too far	36	10%
Refusal of the caregiver to take the child for immunization	35	9%
Other	31	8%

**Health facility access and utilization:** most caregivers (87.8%) accessed immunization services from public facilities, with 84% reported living within a 30-minute distance of a health facility. Despite this proximity, 18% reported challenges when seeking services, including vaccine stockouts (32%) and staff shortages (50%). Caregivers who reported having sought MR services but were not attended to showed a significant association with MR-2 uptake (p = 0.016). Place of delivery was also marginally associated with MR-2 uptake (p = 0.050), with higher uptake among children delivered in health facilities compared to home deliveries. Knowledge-related factors were strongly associated with MR-2 uptake. Caregivers who knew that the MR vaccine is administered in two doses at 9 and 18 months were significantly more likely to have their children receive MR-2 (p < 0.001). Similarly, the point of immunization was significantly associated with uptake (p < 0.001), with higher coverage observed among children vaccinated in public facilities and faith-based organizations. Attendance of antenatal care and distance to the health facility were not significantly associated with MR-2 uptake. Key reasons for missed MR-2 doses were time constraints (33%), fear of vaccine side effects (25%), and long queues at health facilities (22%).

## Discussion

**Statement of principal findings:** this study identified several key determinants of measles-rubella second dose (MR-2) uptake among children aged 18-59 months in Mathare informal settlement. While the overall MR-2 coverage (84%) was higher than national estimates from 2020 (37.2%), uptake varied widely by location, with Ngei Ward recording the lowest at 39%. Religious affiliation and residential ward were significantly associated with MR-2 uptake, while education level approached significance. Although socioeconomic variables such as income and employment were not statistically significant, they emerged as critical barriers in qualitative data. Facility-level factors, including stockouts, staff shortages, and poor communication, contributed to missed opportunities and service gaps.

**Strengths and weaknesses of the study:** a major strength of this study is the mixed-methods approach, which allowed triangulation of quantitative findings with in-depth qualitative insights. The use of multi-stage sampling enhanced representativeness across diverse wards in Mathare. The high response rate (103%) also strengthens the validity of the results. However, limitations include potential recall bias in reporting vaccination status, although this was mitigated by verification using vaccination cards.

**Comparison with existing literature:** the association between caregiver education and higher MR-2 uptake observed in this study is consistent with previous research demonstrating that caregiver education is positively associated with vaccination uptake due to enhanced health literacy, and improved understanding of immunization schedules [7]. However, most of the existing literature in low-income urban settings focuses primarily on uptake of the first dose of Measles-Containing Vaccine (MCV1), with limited attention to completion of the second dose, particularly in informal settlements. This study contributes by specifically examining MR-2 uptake, an increasingly critical indicator for measles and rubella elimination.

Similarly, concerns related to vaccine safety, misinformation and fear of side effects identified in this study echo global and local studies that identify these factors as core drivers of vaccine hesitancy [8,9]. Previous studies found that younger or less-educated caregivers are more susceptible to misinformation and less likely to adhere to immunization schedules [10]. This study's findings build on existing evidence by demonstrating how individual caregiver factors interact with contextual challenges in informal settlements, highlighting the need for targeted communication strategies beyond general health education.

Although quantitative analysis in this study did not show a statistically significant association between household economic indicators and MR-2 uptake, qualitative findings highlighted economic constraints, such as competing household demands and transport costs, as important barriers. This difference highlights a limitation in previous studies that rely solely on quantitative measures and fail to capture lived experiences influencing vaccination behavior. This study adds depth to existing evidence and highlights the value of mixed-methods approaches in understanding immunization gaps in informal urban settings.

Like previous studies in urban slums in Kenya and Nigeria [11,12]. This study also identified health system inefficiencies, including irregular vaccine supply and long wait times and missed opportunities during facility visits, as critical barriers to completion of routine immunization schedules. However, earlier studies often examine these barriers in isolation, without linking them explicitly to MR-2 uptake or exploring their combined effect with caregiver-level determinants. This study addresses this gap by simultaneously examining demographic, socio-economic, and health system factors within a single analytical framework.

**Implications for immunization practice and policy:** the study highlights the need for coordinated strategies to improve MR-2 uptake in informal settlements. Health workers and community health volunteers should provide targeted education, address misconceptions, and reinforce follow-up after the first MR dose. Facilities should optimize staffing, reduce waiting times, and ensure consistent vaccine availability. At the policy level, counties and the Ministry of Health should strengthen supply chain management, integrate reminder and defaulter-tracking systems, and support outreach programs tailored to the realities of urban informal settings. Together, these measures can reduce missed opportunities, close immunity gaps, and contribute to measles and rubella elimination goals.

**Generalizability of findings:** although this study was conducted in Mathare Sub-County, the findings are likely applicable to other urban informal settlements with similar socio-economic and health system characteristics. Many informal settlements in Kenya and other low- and middle-income countries face comparable challenges, including high population density, limited access to health services, caregiver knowledge gaps, and health system constraints such as long waiting times and vaccine stock-outs. Therefore, the identified demographic, socio-economic, and health system-related determinants of MR-2 uptake may be relevant to similar settings, while recognizing that local contextual differences may influence the relative importance of specific factors.

**Unanswered questions and future research:** future studies should investigate the long-term impact of targeted interventions such as mobile reminders and community health promoter engagement. Additionally, a longitudinal study could explore how caregiver knowledge and attitudes evolve and affect completion of vaccine schedules. Further exploration is also needed on the role of digital tools in reducing missed opportunities in immunization delivery.

**Limitations:** the research faced several constraints, including inadequate data management at the facility level and recall bias, as caregivers were mostly unable to accurately recall information about the child's immunization routine. To address these challenges, we utilized desktop reviews and consulted various Ministry of Health (MOH) reporting tools. These included MOH form 510-children's immunization records, MOH form 511- register for Child Welfare Clinic (CWC), MOH form 710-immunization services uptake, MOH 702 immunization tally sheet, MOH 711- integrated reproductive and child health summary and the MOH 216- mother and child health booklet.

**Recommendations:** improving MR-2 uptake in informal settlements such as Mathare requires coordinated short- and long-term actions. In the short term, ensuring consistent vaccine availability, optimizing staffing levels, and reducing waiting times at health facilities are essential to minimize missed opportunities. Strengthening interpersonal communication between the community members, health workers and community health volunteers can help address misinformation and increase awareness of the MR-2 schedule.

In the medium term, increasing outreach strategies, including mobile clinics, extended clinic hours for immunization services, and community-based follow-up by CHVs, can improve access for caregivers facing economic challenges and time constraints. Further, integrating an SMS-based reminder system into existing health information sharing platforms may further support adherence to immunization schedules.

In the long term, investments in health system strengthening, improved data management, and continuous operational research are needed to sustain gains in MR-2 coverage. Engaging community leaders and promoting male involvement in child health decision-making may also contribute to improved vaccine acceptance and completion.

## Conclusion

This study found that the uptake of the second dose of the measles-rubella vaccine (MR-2) among children aged 18-59 months in Mathare informal settlement was 84%, with substantial intra-settlement variation across wards, dropping as low as 39% in Ngei Ward. Key factors influencing MR-2 uptake included caregiver education, religious affiliation, knowledge of the immunization schedule, and health system issues such as vaccine stockouts, staff shortages, and long waiting times. Economic constraints, transport costs, competing caregiver responsibilities, misinformation, and fear of side effects also contributed to missed vaccinations. The drop between the first and second doses highlights gaps in caregiver follow-up and health system support. Strengthening follow-up between MR-1 and MR-2, improving service delivery, and using community-based interventions are essential to close immunity gaps and increase coverage in informal urban settings.

### What is known about this topic

- Uptake of the second dose of measles-rubella vaccine (MR-2) is often lower than the first dose, particularly in underserved communities;
- Urban informal settlements present unique barriers to immunization, including both service-related and socio-economic factors;
- Misinformation and negative healthcare experiences are known to contribute to vaccine hesitancy.

### What this study adds

- The study shows large differences in MR-2 uptake within one informal settlement, highlighting the need for tailored, local solutions;
- It combines quantitative and qualitative evidence to explain why some children miss their second dose, ranging from service delivery issues to caregiver beliefs and daily struggles;
- The research outlines actionable recommendations, such as using community health volunteers, mobile reminders, and outreach strategies tailored to the realities of informal settlements.
